# Serum levels of pancreatic stone protein (PSP)/reg1A as an indicator of beta-cell apoptosis suggest an increased apoptosis rate in hepatocyte nuclear factor 1 alpha (HNF1A-MODY) carriers from the third decade of life onward

**DOI:** 10.1186/1472-6823-12-13

**Published:** 2012-07-18

**Authors:** Siobhan Bacon, Ma Peyh Kyithar, Jasmin Schmid, Syed R Rizvi, Caroline Bonner, Rolf Graf, Jochen HM Prehn, Maria M Byrne

**Affiliations:** 1 Department of Diabetes, Mater Misericordiae University Hospital, Dublin 7, Ireland; 2 Department of Physiology and Medical Physics, Centre for Systems Medicine, Royal College of Surgeons in Ireland, Dublin 2, Ireland; 3 Department of Visceral and Transplantation Surgery, University Hospital Zurich, Zurich, Switzerland; 4 Department of Endocrinology, Mater Misericordiae University Hospital, 30 Eccles Street, Dublin 7, Ireland

**Keywords:** Maturity onset diabetes of the young (MODY), Apoptosis, Serum biomarker, Beta-Cell, Type 1 diabetes, Pancreatic stone protein (PSP), Regenerating gene 1A (reg1A)

## Abstract

**Background:**

Mutations in the transcription factor hepatocyte nuclear factor-1-alpha (HNF1A) result in the commonest type of maturity onset diabetes of the young (MODY). HNF1A-MODY carriers have reduced pancreatic beta cell mass, partially due to an increased rate of apoptosis. To date, it has not been possible to determine when apoptosis is occurring in HNF1A-MODY.We have recently demonstrated that beta cell apoptosis stimulates the expression of the pancreatic stone protein/regenerating (PSP/reg) gene in surviving neighbour cells, and that PSP/reg1A protein is subsequently secreted from these cells. The objective of this study was to determine whether serum levels of PSP/reg1A are elevated during disease progression in HNF1A-MODY carriers, and whether it may provide information regarding the onset of beta-cell apoptosis.

**Methods:**

We analysed serum PSP/reg1A levels and correlated with clinical and biochemical parameters in subjects with HNF1A-MODY, glucokinase (GCK-MODY), and type 1 diabetes mellitus. A control group of normoglycaemic subjects was also analysed.

**Results:**

PSP/reg1A serum levels were significantly elevated in HNF1A-MODY (n = 37) subjects compared to controls (n = 60) (median = 12.50 ng/ml, IQR = 10.61-17.87 ng/ml versus median = 10.72 ng/ml, IQR = 8.94-12.54 ng/ml, p = 0.0008). PSP/reg1A correlated negatively with insulin levels during OGTT, (rho = −0.40, p = 0.02). Interestingly we noted a significant positive correlation of PSP/reg1A with age of the HNF1A-MODY carriers (rho = 0.40 p = 0.02) with an age of 25 years separating carriers with low and high PSP/reg1A levels. Patients with type 1 diabetes mellitus also had elevated serum levels of PSP/reg1A compared to controls, however this was independent of the duration of diabetes.

**Conclusion:**

Our data suggest that beta cell apoptosis contributes increasingly to the pathophysiology of HNF1A-MODY in patients 25 years and over. PSP/reg1A may be developed as a serum marker to detect increased beta-cell apoptosis, or its therapeutic response.

## Background

Maturity-onset diabetes of the young (MODY), is a monogenic form of diabetes characterized by autosomal dominant mode of inheritance including a three-generation family history of diabetes, age at diagnosis of diabetes of 25 years or less in at least one family member and reduced glucose-stimulated insulin secretion [1]. MODY can result from mutations in at least six different genes [2]. The two most common forms are HNF1A-MODY and glucokinase (GCK)-MODY in all populations studied accounting for approximately 70% of all cases [3].

Insulin secretory defects have been observed in carriers of HNF1A-MODY and GCK-MODY with and without diabetes [4,5]. HNF1A encodes a transcription factor important for pancreatic development, beta cell differentiation and function. HNF1A-MODY subjects with pre-diabetes have defective glucose-induced beta cell insulin secretion indicative of reduced beta cell mass [4]. Homozygous *hnf-1a/tcf-1* knockout mice likewise have a reduced beta cell mass [6]. HNF1A-MODY is associated with a severe and progressive clinical course with up to 50% of patients requiring insulin. The decline in functional beta cell mass may be attributed to increased beta cell apoptosis [7-9]. Mutations in HNF1A are usually detected later in life when they are incidentally discovered through a screening programme or if subjects become symptomatic. Diabetes usually becomes manifest when additional superimposed environmental factors supervene such as a physiological decrease in insulin sensitivity with puberty and pregnancy. The decline in beta cell mass therefore, is progressive but gradual. The possible contribution of apoptosis to this decrease in beta cell mass however, has not been investigated due to a lack of accessible, specific biomarkers for beta cell apoptosis.

In contrast to HNF1A-MODY carriers, patients with GCK-MODY do not show a comparably progressive decrease in beta cell mass. Glucokinase is a key regulatory enzyme in the pancreatic beta cell catalyzing the conversion of glucose to glucose-6-phosphate, the first step in glycogen storage and glycolysis. GCK-MODY is often subclinical but can be detected at any stage of life [10,11]. GCK-MODY carriers have mild fasting and post-prandial hyperglycaemia from birth, lack of progression, and absence of insulin requirement and vascular complications [10-12]. Patients with GCK mutations, in contrast to HNF1A-MODY rarely require any pharmacological intervention and the majority are managed with diet alone.

One of the challenges in evaluating the contribution of beta cell apoptosis in human disease development is the lack of appropriate tools to evaluate the extent of beta cell apoptosis in patients other than *post mortem* studies [13]. Another complicating factor is that the actual process of apoptosis execution is very rapid and occurs within minutes [14], and apoptosing cells can rapidly detach from the extracellular matrix, and are subsequently phagocytised [15], thereby limiting the chance of detecting apoptosis by *in-vivo* imaging techniques. An alternative strategy therefore, is to detect signals that are generated directly or indirectly from apoptosing beta cells. Previous studies from our laboratory have demonstrated that beta cells undergoing apoptosis induce the expression of the PSP/*reg* gene in neighbouring beta cells. This paracrine gene induction occurs in a caspase-dependent manner, and is mediated by the shedding of microparticles from apoptosing cells. PSP/reg1A protein is subsequently secreted from neighbouring cells, and provides a regenerative cue to the microenvironment. We also demonstrated elevated serum levels of PSP/reg1A, the human homologue of PSP/reg, in human subjects with HNF1A-MODY and in subjects with type 1 diabetes mellitus, suggesting that the endocrine pancreas is the primary source of this signal [16].

In the present study, we sought to clinically validate this finding with a larger group and to investigate whether elevated PSP/reg1A levels in HNF1A-MODY correlated with a clinical phenotype and progression of disease. We also investigated whether PSP/reg1A was upregulated in GCK-MODY.

## Methods

### Subjects

37 HNF1A-MODY and 13 patients with GCK-MODY participated in the study. The HNF1A-MODY group consisted of 13 different families and the GCK-MODY group represented 6 different families. HNF1A mutations included L17H, G207D, P291finsC, S352fsdelG, F426X, P379T, and IVS7-6 G > A, and R200Q/N. GCK mutations included D160N, Y61X, A378V, L146fs, Ile293Arg, and p.Asp311fs. A historic value for PSP/reg1A was also available based on data from 60 normoglycaemic subjects (median = 10.72 ng/ml, IQR = 8.94-12.54 ng/ml). In addition, 27 patients with antibody positive (GAD and or Islet cell antibody) type 1 diabetes mellitus were recruited from the diabetes outpatients in the Mater Misericordiae University Hospital. Clinically, on presentation, all subjects with type 1 diabetes mellitus had osmotic symptoms with ketosis requiring insulin from diagnosis. There was no significant family history recorded in any of the subjects with type 1 diabetes mellitus. All subjects were BMI-matched. The clinical characteristics of all groups analyzed are presented in Table 1 and Table 2 contains the glucose levels during OGTT in HNF1A-MODY subjects. Ethics approval was attained from the ethics committee at the Mater Misericordiae University Hospital. All study subjects gave written informed consent to participate in the study.

**Table 1:** Clinical characteristics of subjects

**Subject group**	**HNF1A-MODY**	**Glucokinase Mutations**	**Type 1 diabetes mellitus**
**Number of subjects**	37	13	27
**Age (yrs)**	43 (21–52)	38 (27–60)	41 (23–52)
**Diabetes duration (yrs)**	6 (3–19)	From birth	21 (3–34)
**BMI (kg/m**^**2**^**)**	24.4 (22.0-26.3)	24.0 (21.6-26.4)	24.4 (22.3-28.6)
**Fasting plasma glucose (mmol/l)**	6.8 (5.4-9.0)	6.5 (6.0-7.0)	9.6 (8.5-11.1)
**Fasting C peptide (ng/ml)**	1.5 (1.0-1.8)	1.3 (1.0-1.7)	<0.5^a^
**HbA**_**1c**_**(%)**	7.0 (6.3-7.9)	6.3 (6.1-6.6)	7.7 (7.2-9.2)

Clinical characteristics of subjects with HNF1A-MODY, GCK-MODY, type 1 diabetes mellitus,

^a^ = median is below detection limit. Data are given as median (IQR).

**Table 2:** Glucose levels during OGTT in HNF1A-MODY subjects

**Time (mins)**	**Glucose (mmol/l)**
0	7.6 ± 0.5
30	13.3 ± 0.9
60	17.7 ± 1.5
90	18.3 ± 1.5
120	18.6 ± 1.9

Data are presented as mean and standard error of the mean (SEM).

### Clinical and laboratory measurements

All subjects underwent a full clinical assessment, including a full medical history and physical examination. Anthropometric measurements including weight, height, and body mass index (BMI) were obtained. Blood samples were drawn for the measurement of HBA_1c_, fasting lipids, full blood count, thyroid function, renal and liver profiles, glutamic acid decarboxylase (GAD65) auto antibodies, and pancreatic islet cell auto antibodies (ICA). In addition a blood sample for PSP/reg1A was drawn.

A 75 g OGTT was performed on subjects (excluding subjects with type 1 diabetes mellitus) after a 12-h overnight fast with measurement of glucose, insulin and C-peptide at baseline and at 30 minute intervals for 120 minutes to determine the degree of glucose tolerance and insulin secretory response. In patients with diabetes, oral hypoglycaemic agents were stopped at least 48 h before the OGTT while, in those taking insulin, long-acting insulin therapy was stopped for 24-h and short-acting insulin stopped for 12-h prior to OGTT. The diagnostic criteria for the American Diabetes Association was used to define the degree of glucose tolerance.

#### Assays

All laboratory analyses were performed with commercially available standardized methods. The plasma glucose concentration was measured using Beckman Synchron DXC800 (Beckman Instruments Inc, Brea, USA). HbA_1c_ was determined using high performance liquid chromatography (Menarini HA81-10, Rome, Italy). Insulin and C-peptide were analyzed using Immulite 2000 immunoassay (Siemens Healthcare Diagnostics, Deerfield, IL, USA). GAD antibodies were analysed using competitive fluid-phase radioimmunoassay by the neurosciences group in John Radcliffe Hospital in Oxford, and ICA by indirect immunofluoresence test by the Supra-Regional Protein Reference Unit and Department of Immunology in Sheffield, UK.

The ELISA to quantify human PSP/reg was performed using the anti-sera from rabbits and guinea pigs immunized with recombinant human PSP/reg protein as previously described [17,18]. Patient serum PSP/reg levels were compared with standard amounts of protein of recombinant human PSP/reg. The technical specificity of the PSP/reg assay has been determined: addition of 0.5 ng/ml to diluted serum from different individuals gave a recovery of 101 +/− 20% (n = 19). The intra-plate and inter-plate variability is less than 5% and 10% respectively.

### Statistical analysis

Data are presented as median and interquartile range (IQR). Areas under the curve (AUCs) for insulin were calculated using the trapezoidal rule. Statistical analysis was performed using MATLAB (Mathworks, Natick, Massachusetts, USA). Differences between groups were determined by two-sided Mann–Whitney/Wilcoxon rank sum test. The Spearman correlation test was used for correlation analysis. Differences and correlations were considered to be significant at *P* < 0.05.

## Results

### PSP/reg1A serum levels are elevated in HNF1A-MODY subjects and correlate negatively with AUC insulin

**Figure 1:**
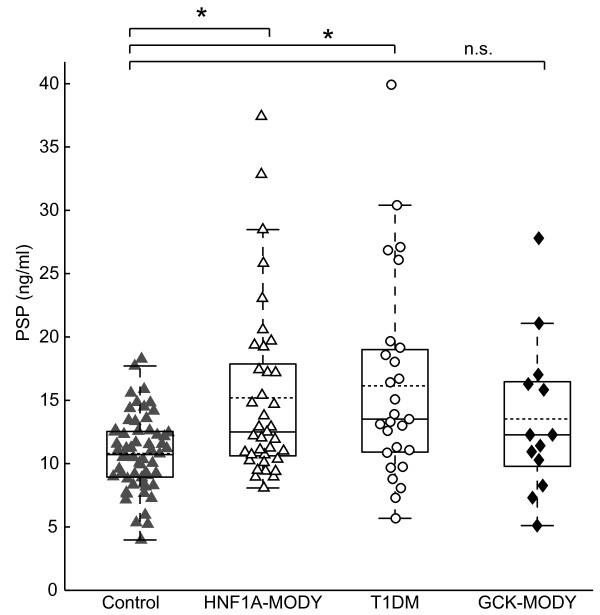
** Serum levels of PSP/reg1A in HNF1A-MODY(Δ), T1DM (○) and GCK-MODY (♦) versus non-diabetic controls (▴).** Solid lines/box plot showing median and interquartile range (IQR) and dotted line showing mean. Serum levels of PSP/reg1A in HNF1A-MODY are at a median of 12.50 ng/ml showing an IQR of 10.61 to 17.87 ng/ml in n = 37 patients. The median in n = 27 type 1 diabetes mellitus (T1DM) patients is 13.52 ng/ml with IQR of 10.91 to 19.01 ng/ml and the median in n = 60 control subjects is 10.72 ng/ml and IQR is 8.94-12.54 ng/ml. A Mann–Whitney *U*-test of the groups resulted in (*) p = 0.0008 for differences between HNF1A-MODY and controls and (*) p = 0.0007 for comparison of T1DM versus controls. The median of GCK-MODY is 12.27 ng/ml with an IQR of 9.79 to 16.47 ng/ml in n = 13 patients, which resulted in (n.s.) p = 0.164 when compared to controls.

We determined the fasting serum PSP/reg1A levels in the HNF1A-MODY group using a previously described ELISA assay [17,18]. The levels of PSP/reg1A were significantly elevated in subjects with HNF1A-MODY compared to historic controls (median = 12.5 ng/ml, IQR = 10.61-17.87 ng/ml versus median = 10.72 ng/ml, IQR = 8.94-12.54 ng/ml, p = 0.0008, Figure 1). These results validate our previous finding that PSP/reg1A, produced in response to beta cell apoptosis, is elevated in HNF1A-MODY [16].

**Figure 2:**
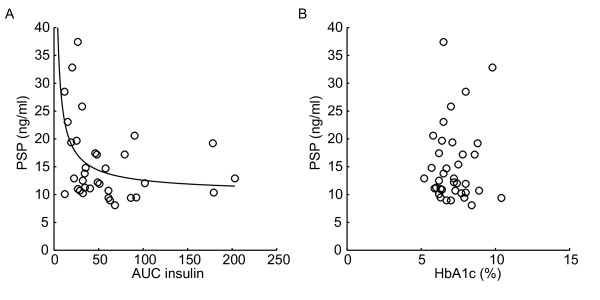
** Correlations of PSP/reg1A in HNF1A-MODY.** (**A**) PSP/reg1A correlation with AUC of insulin (Spearman r = −0.40, p = 0.02, n = 34)**.** The regression curve highlights the negative correlation (R^2^ = 0.17). (**B**) No correlation was found between PSP/reg1A and HbA_1c_ (Spearman r = −0.09, p = 0.62, n = 37).

We next sought to ascertain the presence or absence of a correlation between levels of PSP/reg1A and insulin levels determined using a 120 minute OGTT and calculated AUC insulin using trapezoidal rule in subjects with HNF1A-MODY. We found a negative correlation between AUC insulin and PSP/reg1A (rho = −0.40, p = 0.02, Figure 2A).

### PSP/reg1A levels do not correlate with HbA_1c,_ triglyceride levels or clinical infectious/inflammatory markers

Potential factors leading to beta cell death in diabetes include high concentrations of glucose [19]. Surprisingly, however, we did not find a correlation between PSP/reg1A and HbA_1c_ (rho = −0.09, p = 0.62, Figure 2B). There was, likewise no correlation noted with total cholesterol (rho = 0.11, p = 0.58), HDL cholesterol (rho = 0.12, p = 0.54), LDL cholesterol (rho = 0.05, p = 0.78) or triglycerides (rho = −0.21, p = 0.25). It has also been postulated that PSP may be increased during infection/inflammation [20]. However, we did not detect any correlation with common markers of inflammation such as total white cell count (rho = 0.30, p = 0.09) or neutrophil count (rho = 0.30, p = 0.09).

### PSP/reg1A serum level increase with age in HNF1A-MODY subjects

**Figure 3:**
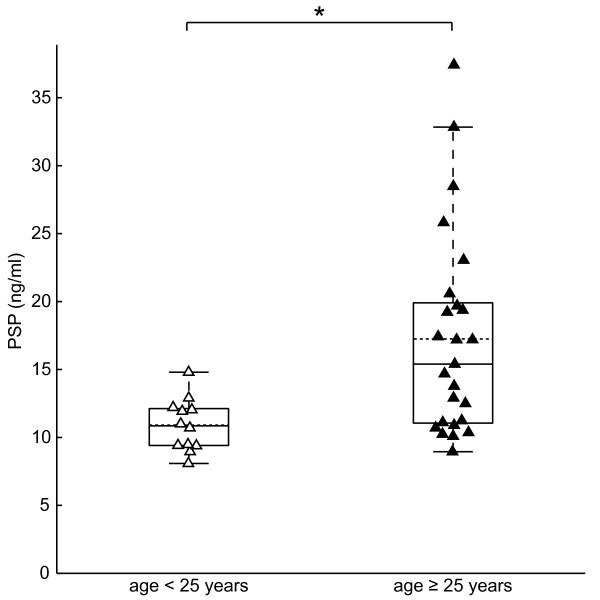
**Serum levels of PSP/reg1A at age < 25 years (Δ) versus older (▴) in HNF1A-MODY subjects.** Solid lines/box plot showing median and interquartile range and dotted line showing mean. The medians of PSP/reg1A are demonstrated between young HNF1A-MODY (median = 10.85 ng/ml, IQR = 9.41-12.12 ng/ml, n = 12) and older subjects (median = 15.40 ng/ml, IQR = 11.05-19.91 ng/ml, n = 25), Mann–Whitney *U*-test (*) p = 0.004.

As beta cell dysfunction progresses in HNF1A-MODY patients [21], we next explored whether there was a correlation between PSP/reg1A serum levels and age. We found a positive correlation between age and PSP/reg1A (r = 0.40, p = 0.02). Further analysis of the data demonstrated that those diagnosed with HNF1A-MODY under 25 years of age had significantly lower levels of PSP/reg1A than those diagnosed at the age of 25 years or over (median = 10.85 ng/ml, IQR = 9.41-12.12 ng/ml versus median = 15.40 ng/ml, IQR = 11.05-19.91 ng/ml, p = 0.0039, Figure 3).

### PSP/reg1A serum levels are elevated in autoantibody-type 1 diabetes mellitus, but independent of age and disease onset

We next wished to confirm these data in a second group of patients with the principal pathogenic factor being beta cell destruction. A cohort of auto-antibody positive, BMI-matched type 1 diabetes mellitus patients were compared with the (n = 60) historic controls. We found that PSP/reg1A levels were significantly higher in the type 1 diabetes mellitus population (median = 13.52 ng/ml, IQR = 10.91-19.01 ng/ml versus median = 10.72 ng/ml, IQR = 8.94-12.54 ng/ml, p = 0.0007, Figure 1). Interestingly, we did not detect any correlation between age (rho = 0.27, p = 0.18) or disease onset (rho = 0.33, p = 0.09) and PSP/reg1A serum levels in this cohort of patients, suggesting that beta cell apoptosis occurs during disease independent of these factors. As with the subjects with HNF1A-MODY there was no correlation between PSP/reg1A levels and HbA_1c_ (rho = −0.08, p = 0.68). There was likewise, no correlation with total cholesterol (rho = −0.08, p = 0.75), HDL cholesterol (rho = 0.24, p = 0.32), LDL cholesterol (rho = −0.31, p = 0.19), triglycerides (rho = −0.07, p = 0.77), or white cell count (rho = −0.22, p = 0.39).

### PSP/reg1A serum levels are not significantly elevated in GCK-MODY

We next tested whether patients with GCK-MODY also showed a pronounced increase in PSP/reg1A levels. Although there was a tendency towards elevated PSP/reg1A levels, we did not find a statistically significant elevation of PSP/reg1A level when compared to historic controls (median = 12.27 ng/ml, IQR = 9.79-16.47 ng/ml versus median = 10.72 ng/ml, IQR = 8.94-12.54 ng/ml, p = 0.16, Figure 1). There was also no correlation noted with AUC insulin (r = 0.21, p = 0.55) or HbA_1c_ (r = 0.52, p = 0.07)_._ There was no statistically significant difference between serum PSP/reg1A levels of GCK-MODY and HNF1A-MODY or type 1 diabetes mellitus patients (p = 0.55/p = 0.34 respectively).

## Discussion

The Irish HNF1A-MODY cohort investigated in this study has been recently characterized [22]. This study reported on a mutation identification rate of 30.5% among Irish adults clinically selected for HNF1A-MODY from attendees at the diabetes clinic. In the present study, we report that PSP/reg1A levels are significantly elevated in human subjects with HNF1A-MODY when compared to controls. Higher levels of circulating PSP/reg1A in HNF1A-MODY were associated with increased age suggesting that the rate of beta cell apoptosis increases during disease progression and within the third life decade. PSP/reg1A did not correlate with HbA_1c_ nor did it correlate with the common clinical infectious/inflammatory markers. Patients with type 1 diabetes mellitus also showed elevated PSP/reg1A levels independent of age or disease onset. Together these data suggest that PSP/reg1A may be a clinical indicator of beta cell apoptosis.

We have previously provided biological evidence in insulinoma cell lines and transgenic mice models of HNF1A-MODY, that beta cells undergoing apoptosis induce the expression of the PSP/reg gene in neighbouring beta cells [16]. The induction of PSP/reg during apoptosis was inhibited in cells treated with caspase inhibitors. Paraffin embedded pancreatic sections from 5 month old diabetic mice expressing HNF1A-MODY in beta cells also demonstrated elevated expression of PSP/reg throughout the islets compared with wild-type mice, with PSP/reg positive cells positioned in the vicinity of cells displaying apoptotic nuclear morphology. These earlier findings demonstrated that PSP/reg1A gene expression was induced during apoptosis in in vitro and animal models of diabetes. One of the core findings of the present study is that PSP/reg1A serum levels may be used as a non-invasive marker to evaluate the extent of beta cell apoptosis during disease progression in human subjects.

The development and implementation of non-invasive techniques for the quantitative measurement of beta cell apoptosis would help in the early diagnosis of beta cell dysfunction in pre-clinical phases of diabetes. Furthermore, it would enable evaluation of emerging therapeutic approaches which focus on preservation of beta cell mass through stimulation of anti-apoptotic signalling. Currently, only indirect methods that evaluate total beta cell mass through secretory responses of islet cells are available, such as arginine-induced insulin secretion [23,24]. These rely on measuring insulin secretion following different metabolic stimuli. Direct visualization of either native or transplanted pancreatic islets was unsuccessful due to their small size, the little difference in physical characteristics from the surrounding tissue, and their relatively low number dispersed over a large area of the pancreas or the liver as the most common site of transplantation [25]. However, modern diagnostic equipment may provide very high sensitivity by positron emission tomography (PET) and single photon emission computed tomography (SPECT), spatial resolution by magnetic resonance imaging (MRI), or both (by PET/CT) [26]. Despite these advances in imaging devices, the main problem is the lack of a specific structural or molecular marker to enable differentiation between scattered islets or single beta cells, and surrounding tissue. Moreover, these techniques require dedicated imaging centres and are costly.

A previously reported paper hypothesized as to whether serum reg protein levels could be representative of the regenerative process at the beta cell level during the early phases of type 1 diabetes mellitus in humans [27]. We have found that PSP/reg1A levels are higher in type 1 diabetes mellitus but independent of disease onset or age. This finding should be considered in the context of recent investigations suggesting that beta cell regeneration may occur even in patients with long standing type 1 diabetes mellitus [28-31]. Our study indicates that this regeneration may be accompanied by continuing beta cell apoptosis, even in patients with long standing type 1 diabetes mellitus. Therefore, therapeutic interventions that boost beta cell survival could be a valuable therapeutic approach even in long standing type 1 diabetes mellitus.

In contrast to type 1 diabetes mellitus patients who have a rapid disease onset, HNF1A-MODY carriers represent an interesting and important study group for the study of beta cell apoptosis during disease progression as carriers will ultimately develop diabetes [1]. As demonstrated in HNF1A-MODY subjects’ beta cell apoptosis appeared to correlate with age, but not with HbA_1c_ suggesting that aging is an important risk factor, and that young islets have an increased resistance towards mutant HNF1A-induced apoptosis. Although patients with a GCK-MODY mutation did not have significantly elevated levels of serum PSP/reg1A when compared to controls, we cannot draw any conclusions in relation to this group due to the limited numbers available. There was also no statistically significant difference between serum PSP/reg1A levels of GCK-MODY and HNF1A-MODY or type 1 diabetes mellitus patients.

The pancreatic acinar cells are considered to be the most important source of PSP/reg under normal conditions however in pathological conditions other cells/tissues may contribute to elevated levels of the protein. PSP has been shown to be elevated in liver/pancreatic disease and in chronic renal failure [32]. When originally discovered, PSP/reg was proposed to be a marker of pancreatic injury in pancreatitis [33,34] however; subsequent studies have failed to demonstrate elevated levels even after severe pancreatitis [35]. In this study the subjects had normal liver and renal function. Furthermore, PSP/reg1A levels did not correlate with infection/inflammation markers such as white cell count.

As a result of the growing interest in beta cell preservation as a potential cure for diabetes, a number of different treatments aimed at protecting the beta cell and maintaining beta cell mass have been evaluated. Insulinotropic agents such as repaglinide or GLP-1 have been shown to be cytoprotective *in-vitro* and in animal models of diabetes [36,37]. From a clinical aspect, beta cell protection may also be induced by reducing the peripheral insulin demand by either improving sensitivity e.g. through physical exercise, pharmacologically using metformin/glitazones, or by lowering blood glucose through the administration of exogenous insulin [38-40]. Our study suggests that PSP/reg1A serum levels may be useful as a biomarker for such potentially cytoprotective treatment paradigms.

## Conclusion

PSP/reg1A may be developed as a serum marker to detect alterations in the rate of beta cell apoptosis during disease or in response to treatment. Furthermore, our data suggests that beta cell apoptosis contributes to the pathophysiology of HNF1A-MODY in patients 25 years and over.

## Abbreviations

AUC, Area under the curve; GCK, Glucokinase; GAD65, Glutamic acid decarboxylase; HNF, Hepatocyte nuclear factor; ICA, Islet cell antibodies; OGTT, Oral glucose tolerance test; PSP, Pancreatic stone protein; TG, Triglycerides.

## Competing interests

The authors declare that they have no competing interests.

## Authors’ contributions

SB coordinated and contributed to the recruitment of subjects, carried out functional studies, interpreted results and wrote manuscript, PMK contributed to the recruitment of subjects and carried out functional studies, JS performed statistical analysis of data, SRR contributed to the recruitment of subjects and carried out functional studies. RG carried out the PSP/reg assay. CB contributed to the writing of the manuscript and performed initial basic studies, JHM contributed to the interpretation of the results, the writing and the critical review of the manuscript. MMB coordinated and designed the study, contributed to the interpretation of the results, the writing and the critical review of the manuscript. All authors read and approved the final manuscript.

## Pre-publication history

The pre-publication history for this paper can be accessed here:

http://www.biomedcentral.com/1472-6823/12/13/prepub

## References

[B1] Fajans SS Bell GI Polonsky KS Molecular mechanisms and clinical pathophysiology of maturity-onset diabetes of the young N Engl J Med 2001 345 971 980 10.1056/NEJMra002168 11575290

[B2] Fajans SS Bell GI MODY: history, genetics, pathophysiology, and clinical decision making Diabetes Care 2011 34 1878 1884 10.2337/dc11-0035 21788644 PMC3142024

[B3] Ellard S Bellanne-Chantelot C Hattersley AT Best practice guidelines for the molecular genetic diagnosis of maturity-onset diabetes of the young Diabetologia 2008 51 546 553 10.1007/s00125-008-0942-y 18297260 PMC2270360

[B4] Byrne MM Sturis J Menzel S Yamagata K Fajans SS Dronsfield MJ Bain SC Hattersley AT Velho G Froguel P Altered insulin secretory responses to glucose in diabetic and nondiabetic subjects with mutations in the diabetes susceptibility gene MODY3 on chromosome 12 Diabetes 1996 45 1503 1510 10.2337/diabetes.45.11.1503 8866553

[B5] Byrne MM Sturis J Clement K Vionnet N Pueyo ME Stoffel M Takeda J Passa P Cohen D Bell GI Insulin secretory abnormalities in subjects with hyperglycemia due to glucokinase mutations J Clin Invest 1994 93 1120 1130 10.1172/JCI117064 8132752 PMC294056

[B6] Pontoglio M Sreenan S Roe M Pugh W Ostrega D Doyen A Pick AJ Baldwin A Velho G Froguel P Defective insulin secretion in hepatocyte nuclear factor 1alpha-deficient mice J Clin Invest 1998 101 2215 2222 10.1172/JCI2548 9593777 PMC508809

[B7] Wobser H Dussmann H Kogel D Wang H Reimertz C Wollheim CB Byrne MM Prehn JH Dominant-negative suppression of HNF-1 alpha results in mitochondrial dysfunction, INS-1 cell apoptosis, and increased sensitivity to ceramide-, but not to high glucose-induced cell death J Biol Chem 2002 277 6413 6421 10.1074/jbc.M108390200 11724785

[B8] Wobser H Bonner C Nolan JJ Byrne MM Prehn JH Downregulation of protein kinase B/Akt-1 mediates INS-1 insulinoma cell apoptosis induced by dominant-negative suppression of hepatocyte nuclear factor-1alpha function Diabetologia 2006 49 519 526 10.1007/s00125-005-0119-x 16440211

[B9] Yamagata K Nammo T Moriwaki M Ihara A Iizuka K Yang Q Satoh T Li M Uenaka R Okita K Overexpression of dominant-negative mutant hepatocyte nuclear fctor-1 alpha in pancreatic beta-cells causes abnormal islet architecture with decreased expression of E-cadherin, reduced beta-cell proliferation, and diabetes Diabetes 2002 51 114 123 11756330 10.2337/diabetes.51.1.114

[B10] Froguel P Vaxillaire M Sun F Velho G Zouali H Butel MO Lesage S Vionnet N Clement K Fougerousse F Close linkage of glucokinase locus on chromosome 7p to early-onset non-insulin-dependent diabetes mellitus Nature 1992 356 162 164 10.1038/356162a0 1545870

[B11] Stoffel M Patel P Lo YM Hattersley AT Lucassen AM Page R Bell JI Bell GI Turner RC Wainscoat JS Missense glucokinase mutation in maturity-onset diabetes of the young and mutation screening in late-onset diabetes Nat Genet 1992 2 153 156 10.1038/ng1092-153 1303265

[B12] Cuesta-Munoz AL Tuomi T Cobo-Vuilleumier N Koskela H Odili S Stride A Buettger C Otonkoski T Froguel P Grimsby J Clinical heterogeneity in monogenic diabetes caused by mutations in the glucokinase gene (GCK-MODY) Diabetes Care 2010 33 290 292 10.2337/dc09-0681 19903754 PMC2809268

[B13] Butler AE Janson J Bonner-Weir S Ritzel R Rizza RA Butler PC Beta-cell deficit and increased beta-cell apoptosis in humans with type 2 diabetes Diabetes 2003 52 102 110 10.2337/diabetes.52.1.102 12502499

[B14] Rehm M Dussmann H Janicke RU Tavare JM Kogel D Prehn JH Single-cell fluorescence resonance energy transfer analysis demonstrates that caspase activation during apoptosis is a rapid process. Role of caspase-3 J Biol Chem 2002 277 24506 24514 10.1074/jbc.M110789200 11964393

[B15] Fuchs Y Steller H Programmed cell death in animal development and disease Cell 2011 147 742 758 10.1016/j.cell.2011.10.033 22078876 PMC4511103

[B16] Bonner C Bacon S Concannon CG Rizvi SR Baquie M Farrelly AM Kilbride SM Dussmann H Ward MW Boulanger CM INS-1 cells undergoing caspase-dependent apoptosis enhance the regenerative capacity of neighboring cells Diabetes 2010 59 2799 2808 10.2337/db09-1478 20682686 PMC2963538

[B17] Graf R Schiesser M Lussi A Went P Scheele GA Bimmler D Coordinate regulation of secretory stress proteins (PSP/reg, PAP I, PAP II, and PAP III) in the rat exocrine pancreas during experimental acute pancreatitis J Surg Res 2002 105 136 144 10.1006/jsre.2002.6387 12121700

[B18] Bimmler D Angst E Valeri F Bain M Scheele GA Frick TW Graf R Regulation of PSP/reg in rat pancreas: immediate and steady-state adaptation to different diets Pancreas 1999 19 255 267 10.1097/00006676-199910000-00006 10505756

[B19] Robertson RP Harmon J Tran PO Poitout V Beta-cell glucose toxicity, lipotoxicity, and chronic oxidative stress in type 2 diabetes Diabetes 2004 53 Suppl 1 S119 S124 14749276 10.2337/diabetes.53.2007.s119

[B20] Keel M Harter L Reding T Sun LK Hersberger M Seifert B Bimmler D Graf R Pancreatic stone protein is highly increased during posttraumatic sepsis and activates neutrophil granulocytes Crit Care Med 2009 37 1642 1648 10.1097/CCM.0b013e31819da7d6 19325491

[B21] Shepherd M Shields B Ellard S Rubio-Cabezas O Hattersley AT A genetic diagnosis of HNF1A diabetes alters treatment and improves glycaemic control in the majority of insulin-treated patients Diabet Med 2009 26 437 441 10.1111/j.1464-5491.2009.02690.x 19388975

[B22] Kyithar MP Bacon S Pannu KK Rizvi SR Colclough K Ellard S Byrne MM Identification of HNF1A-MODY and HNF4A-MODY in Irish families: phenotypic characteristics and therapeutic implications Diabetes Metab 2011 37 512 519 10.1016/j.diabet.2011.04.002 21683639

[B23] Teuscher AU Kendall DM Smets YF Leone JP Sutherland DE Robertson RP Successful islet autotransplantation in humans: functional insulin secretory reserve as an estimate of surviving islet cell mass Diabetes 1998 47 324 330 10.2337/diabetes.47.3.324 9519735

[B24] Ryan EA Lakey JR Paty BW Imes S Korbutt GS Kneteman NM Bigam D Rajotte RV Shapiro AM Successful islet transplantation: continued insulin reserve provides long-term glycemic control Diabetes 2002 51 2148 2157 10.2337/diabetes.51.7.2148 12086945

[B25] Evgenov NV Medarova Z Dai G Bonner-Weir S Moore A In vivo imaging of islet transplantation Nat Med 2006 12 144 148 10.1038/nm1316 16380717

[B26] Malaisse WJ On the track to the beta-cell Diabetologia 2001 44 393 406 10.1007/s001250051635 11357468

[B27] Christofilis MA Carrere J Atlan-Gepner C Zevaco-Mattei C Thivolet C Baeza N Figarella C Vialettes B Serum reg protein level is not related to the beta cell destruction/regeneration process during early phases of diabetogenesis in type I diabetes Eur J Endocrinol 1999 141 368 373 10.1530/eje.0.1410368 10526250

[B28] Meier JJ Bhushan A Butler AE Rizza RA Butler PC Sustained beta cell apoptosis in patients with long-standing type 1 diabetes: indirect evidence for islet regeneration? Diabetologia 2005 48 2221 2228 10.1007/s00125-005-1949-2 16205882

[B29] Keenan HA Sun JK Levine J Doria A Aiello LP Eisenbarth G Bonner-Weir S King GL Residual insulin production and pancreatic ss-cell turnover after 50 years of diabetes: Joslin Medalist Study Diabetes 2010 59 2846 2853 10.2337/db10-0676 20699420 PMC2963543

[B30] Lohr M Kloppel G Residual insulin positivity and pancreatic atrophy in relation to duration of chronic type 1 (insulin-dependent) diabetes mellitus and microangiopathy Diabetologia 1987 30 757 762 3322901 10.1007/BF00275740

[B31] Madsbad S Kehlet H Hilsted J Tronier B Discrepancy between plasma C-peptide and insulin response to oral and intravenous glucose Diabetes 1983 32 436 438 10.2337/diabetes.32.5.436 6341127

[B32] Tatemichi N Takahashi C Hayakawa S Hayakawa T Shibata T Kitagawa M Sobajima H Nakae Y Enzyme immunoassay and characterization of pancreatic stone proteins in human urine J Clin Lab Anal 1993 7 365 370 10.1002/jcla.1860070611 8277359

[B33] Hayakawa T Naruse S Kitagawa M Nakae Y Harada H Ochi K Kuno N Kurimoto K Hayakawa S Pancreatic stone protein and lactoferrin in human pancreatic juice in chronic pancreatitis Pancreas 1995 10 137 142 10.1097/00006676-199503000-00005 7716137

[B34] Schmiegel W Burchert M Kalthoff H Roeder C Butzow G Grimm H Kremer B Soehendra N Schreiber HW Thiele HG Immunochemical characterization and quantitative distribution of pancreatic stone protein in sera and pancreatic secretions in pancreatic disorders Gastroenterology 1990 99 1421 1430 1698685 10.1016/0016-5085(90)91171-2

[B35] Jin CX Hayakawa T Ko SB Ishiguro H Kitagawa M Pancreatic stone protein/regenerating protein family in pancreatic and gastrointestinal diseases Intern Med 2011 50 1507 1516 10.2169/internalmedicine.50.5362 21804274

[B36] Farilla L Bulotta A Hirshberg B Li Calzi S Khoury N Noushmehr H Bertolotto C Di Mario U Harlan DM Perfetti R Glucagon-like peptide 1 inhibits cell apoptosis and improves glucose responsiveness of freshly isolated human islets Endocrinology 2003 144 5149 5158 10.1210/en.2003-0323 12960095

[B37] Chen D Liao J Li N Zhou C Liu Q Wang G Zhang R Zhang S Lin L Chen K A nonpeptidic agonist of glucagon-like peptide 1 receptors with efficacy in diabetic db/db mice Proc Natl Acad Sci U S A 2007 104 943 948 10.1073/pnas.0610173104 17213311 PMC1764862

[B38] DeFronzo RA Tripathy D Schwenke DC Banerji M Bray GA Buchanan TA Clement SC Henry RR Hodis HN Kitabchi AE Pioglitazone for diabetes prevention in impaired glucose tolerance N Engl J Med 2011 364 1104 1115 10.1056/NEJMoa1010949 21428766

[B39] Weng J Li Y Xu W Shi L Zhang Q Zhu D Hu Y Zhou Z Yan X Tian H Effect of intensive insulin therapy on beta-cell function and glycaemic control in patients with newly diagnosed type 2 diabetes: a multicentre randomised parallel-group trial Lancet 2008 371 1753 1760 10.1016/S0140-6736(08)60762-X 18502299

[B40] Kahn SE Haffner SM Heise MA Herman WH Holman RR Jones NP Kravitz BG Lachin JM O'Neill MC Zinman B Viberti G Glycemic durability of rosiglitazone, metformin, or glyburide monotherapy N Engl J Med 2006 355 2427 2443 10.1056/NEJMoa066224 17145742

